# High Connectivity in the Deepwater Snapper *Pristipomoides filamentosus* (Lutjanidae) across the Indo-Pacific with Isolation of the Hawaiian Archipelago

**DOI:** 10.1371/journal.pone.0028913

**Published:** 2011-12-22

**Authors:** Michelle R. Gaither, Shelley A. Jones, Christopher Kelley, Stephen J. Newman, Laurie Sorenson, Brian W. Bowen

**Affiliations:** 1 Hawai'i Institute of Marine Biology, University of Hawai'i at Mānoa, Kane'ohe, Hawai'i, United States of America; 2 Human Performance Laboratory, Humboldt State University, Arcata, California, United States of America; 3 Hawai'i Undersea Research Laboratory, University of Hawai'i at Mānoa, Honolulu, Hawai'i, United States of America; 4 Western Australian Fisheries and Marine Research Laboratories, Department of Fisheries, North Beach, Australia; 5 Department of Ecology and Evolutionary Biology, University of California Los Angeles, Los Angeles, California, United States of America; Smithsonian Institution National Zoological Park, United States of America

## Abstract

In the tropical Indo-Pacific, most phylogeographic studies have focused on the shallow-water taxa that inhabit reefs to approximately 30 m depth. Little is known about the large predatory fishes, primarily snappers (subfamily Etelinae) and groupers (subfamily Epinephelinae) that occur at 100–400 m. These long-lived, slow-growing species support fisheries across the Indo-Pacific, yet no comprehensive genetic surveys within this group have been conducted. Here we contribute the first range-wide survey of a deepwater Indo-Pacific snapper, *Pristipomoides filamentosus*, with special focus on Hawai'i. We applied mtDNA cytochrome *b* and 11 microsatellite loci to 26 samples (*N* = 1,222) collected across 17,000 km from Hawai'i to the western Indian Ocean. Results indicate that *P. filamentosus* is a highly dispersive species with low but significant population structure (mtDNA Φ_ST_ = 0.029, microsatellite *F*_ST_ = 0.029) due entirely to the isolation of Hawai'i. No population structure was detected across 14,000 km of the Indo-Pacific from Tonga in the Central Pacific to the Seychelles in the western Indian Ocean, a pattern rarely observed in reef species. Despite a long pelagic phase (60–180 days), interisland dispersal as adults, and extensive gene flow across the Indo-Pacific, *P. filamentosus* is unable to maintain population connectivity with Hawai'i. Coalescent analyses indicate that *P. filamentosus* may have colonized Hawai'i 26 K–52 K y ago against prevailing currents, with dispersal away from Hawai'i dominating migration estimates. *P. filamentosus* harbors low genetic diversity in Hawai'i, a common pattern in marine fishes, and our data indicate a single archipelago-wide stock. However, like the Hawaiian Grouper, *Hyporthodus quernus*, this snapper had several significant pairwise comparisons (*F*_ST_) clustered around the middle of the archipelago (St. Rogatien, Brooks Banks, Gardner) indicating that this region may be isolated or (more likely) receives input from Johnston Atoll to the south.

## Introduction

The effort to understand patterns of genetic connectivity in the Indo-Pacific has largely focused on shallow-water reef associated taxa and pelagic species that support multinational fisheries. The use of molecular tools to identify patterns of population subdivision and the geographic distribution of genetic diversity has provided insights into gene flow and the definition of management units, historical demography, and the impacts of biogeogeographic barriers on dispersal. While there is some incongruence among datasets, large scale patterns have emerged. A growing number of studies indicate a lack of genetic subdivision in reef fishes across nearly 10,000 km from French Polynesia in the Central Pacific to Western Australia and Cocos Keeling in the Indian Ocean [1]–[8], a biogeographic region known as the Indo-Polynesian Province [9]–[11]. Species compositions and phylogeographic analyses indicate that the large spans of open ocean that isolate the Hawaiian Islands are formidable barriers for most shallow-water taxa. Only a subset of the Indo-Pacific shallow-water fauna has successfully colonized Hawai'i and the species that did so are isolated from parent populations as evidenced by the 25% endemism in shallow-water fishes there [12].

Species distributions and a growing number of phylogeographic comparisons of shallow-water species indicate that Pleistocene sea level fluctuations had major impacts on population sizes and connectivity patterns between the Pacific and Indian Oceans. A defining barrier in the Indo-Pacific is the shallow Sunda shelf, surrounding the Malay Peninsula and western islands of Indonesia, and the Sahul shelf off northern Australia and New Guinea. This region, which is exposed during periods of low sea level, separates the Pacific and Indian Oceans and is known as the Indo-Pacific Barrier (IPB) [13]. Over the last 700 K y there have been three to six glacial cycles that lowered sea level as much as 130 m below present levels [14]–[17]. Species on the continental shelves were repeatedly subjected to widespread extirpations and presumably interruption of gene flow between Pacific and Indian Ocean populations. The evidence for the impact of the IPB on shallow-water taxa is extensive and compelling [18].

While our understanding of the impact of biogeographic barriers and historical processes on shallow-water reef organisms is developing, there are no studies that have examined these same processes in deepwater species across the Indo-Pacific. Of particular interest, are the demersal snappers (subfamily Etelinae) found between 100–400 m on the continental shelves and islands throughout the tropical Indo-Pacific and that support important fisheries. While the majority of shallow-water reef species are restricted to a narrow band of habitat in the upper 30 m of tropical oceans it is not unusual for deepwater species to inhabit much wider depth ranges (e.g. 100–300 m). Field studies indicate that shallow-water reef fishes have high site fidelity and move only short distances as adults (<2 km) [19]–[22]. In contrast, some species of deepwater fishes have been recorded traversing channels between islands [23]. Deepwater snappers of the genus *Pristipomoides* tend to live longer and spend more time as pelagic larvae than their shallow-water counterparts [24]. While connectivity patterns in shallow-water taxa have been assessed in many groups and on multiple spatial scales, to our knowledge no Indo-Pacific wide phylogeographic study has been undertaken involving a deepwater species, which begs the question: Do deepwater biota demonstrate different patterns of genetic connectivity than their shallow-water counterparts?

**Figure 1:**
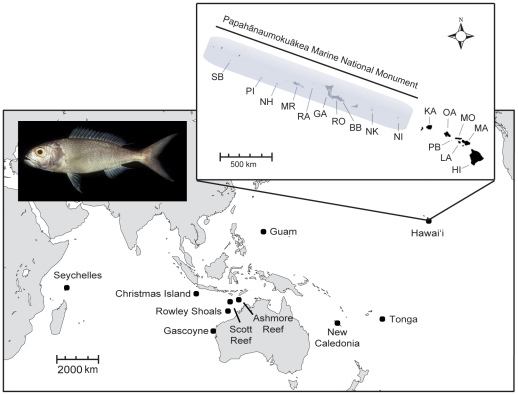
Map of study area. Collection locations for *Pristipomoides filamentosus* including 17 sites within the Hawaiian Archipelago and 9 other locations across the Indo-Pacific. See Table 1 for abbreviations.

Here we present an Indo-Pacific wide survey of a deepwater snapper, the Crimson Jobfish, *Pristipomoides filamentosus* (Valenciennes 1830). This species is better understood than most deepwater fishes due to fishery-oriented research and successful culturing efforts. Adults are found in rocky habitat at depths of 100–360 m from Hawai'i in the Pacific to East Africa and the Red Sea [25], [26] (Fig. 1). *P. filamentosus* is a slow growing and long lived species (≥40 years) [27] that reaches sexual maturity at 3–5 years [28]–[30] and engages in mass spawning of buoyant eggs [31], [32]. Gonadal studies indicate that spawning may occur serially over a protracted period (March to December) [28]. Tagging studies indicate that the majority of adults exhibit restricted movement (0–22 km) while some travel great distances (>400 km) and are able to cross deep water channels [23]. Early life history studies indicate that *P. filamentosus* can remain planktonic at a large size (37–70 mm TL) with a pelagic duration lasting 60–180 days [26], [32]. The length of the early pelagic phase and the ability of some *P. filamentosus* to move great distances as adults indicate that this species may be more dispersive than shallow-water reef associated species. However, this is not a foregone conclusion, as the only other molecular appraisal in this genus (*Pristipomoides multidens*) indicated limited dispersal between Indonesian islands separated by less than 500 km [33]. To test the hypothesis of high dispersal, we employed mitochondrial DNA sequences and 11 microsatellites to assess *P. filamentosus* at nine locations across the Indo-Pacific. Additionally we sampled 17 locations in Hawai'i, a 2,600 km linear array of islands and atolls, to test for fine-scale population connectivity in the archipelago. Specifically we addressed the following questions: 1) Does this deepwater species demonstrate high levels of genetic connectivity across the Indo-Pacific as would be predicted from life history characteristics? 2) Is there gene flow between Hawai'i and Central Pacific populations of *P. filamentosus*? 3) Does Hawai'i contain one or more populations pertinent to management efforts? 4) Does the IPB limit dispersal in this species as has been demonstrated for shallow-water taxa?

## Methods

### Sample collection

A total of 1,222 *P. filamentosus* were collected from 26 locations across the species range including 17 locations within the Hawaiian Archipelago (Fig. 1, Table 1). Samples were obtained either directly from commercial fishers or purchased at local fish markets. Specimens from the Papahānaumokuākea Marine National Monument (Fig. 1) were collected by licensed fishers in the deepwater fishery that closed in 2009. Tissues samples (fin clips or muscle) were preserved in salt-saturated DMSO [34] or in >70% EtOH and stored at room temperature. DNA was isolated using the modified HotSHOT method [35], [36].

**Table 1:** Molecular diversity indices for 26 samples of *Pristipomoides filamentosus*.

	Cyt*b*				Microsatellites				
*Sample Location*	*N*	*N*_H_	*h*	*π*	*N*	*A*	*A*_R_	*H*_O_	*H*_E_
**Hawai'i**									
Hawai'i Island (HI)	75	4	0.13±0.05	0.0003±0.0004	75	5.3	1.95	0.45	0.45
Maui (MA)	79	8	0.26±0.07	0.0006±0.0007	79	5.4	1.99	0.48	0.47
Lana'i (LA)	75	7	0.20±0.06	0.0004±0.0006	73	5.3	1.98	0.44	0.46
Moloka'i (MO)	73	6	0.23±0.07	0.0005±0.0006	74	5.5	2.02	0.48	0.48
Penguin Banks (PB)	29	4	0.26±0.10	0.0006±0.0007	38	4.0	1.98	0.45	0.46
O'ahu (OA)	66	6	0.20±0.07	0.0004±0.0006	64	5.0	1.92	0.42	0.43
Kaua'i (KA)	38	7	0.29±0.10	0.0006±0.0007	37	4.5	1.94	0.43	0.44
Nihoa (NI)	5	1	0.00±0.00	0.0000±0.0000	5	2.5	1.81	0.43	0.43
Necker (NK)	3	2	0.67±0.31	0.0013±0.0017	2	1.7	1.73	0.57	0.60
Brooks Banks (BB)	103	10	0.29±0.06	0.0006±0.0007	95	5.9	1.92	0.42	0.43
St. Rogatien (RO)	71	7	0.24±0.07	0.0005±0.0006	75	5.7	1.99	0.45	0.46
Gardner (GA)	52	7	0.29±0.08	0.0006±0.0007	44	5.1	1.93	0.42	0.43
Raita (RA)	35	5	0.22±0.09	0.0005±0.0006	33	4.5	2.01	0.47	0.47
Maro (MR)	19	2	0.11±0.09	0.0002±0.0004	17	3.8	1.96	0.42	0.45
North Hampton (NH)	11	2	0.18±0.14	0.0004±0.0006	11	3.7	1.94	0.48	0.45
Pioneer (PI)	56	4	0.11±0.06	0.0002±0.0004	53	5.0	1.93	0.45	0.44
Salmon Banks (SB)	8	3	0.46±0.20	0.0010±0.0011	8	2.8	1.95	0.60	0.52
**Indo-Pacific**									
Hawai'i	75	7	0.28±0.08	0.0006±0.0007	75	4.5	1.92	0.49	0.50
Tonga	48	13	0.63±0.08	0.0017±0.0014	38	5.7	2.37	0.51	0.57
New Caledonia	52	15	0.62±0.08	0.0019±0.0014	44	6.3	2.48	0.55	0.60
Guam	7	4	0.71±0.18	0.0017±0.0016	7	3.6	2.59	0.64	0.61
Ashmore Reef	49	16	0.63±0.08	0.0020±0.0015	50	6.2	2.49	0.59	0.59
Gascoyne	44	17	0.70±0.08	0.0021±0.0016	7	2.7	2.15	0.54	0.67
Scott Reef	57	20	0.68±0.07	0.0018±0.0014	62	6.9	2.46	0.52	0.58
Rowley Shoals	75	23	0.59±0.07	0.0018±0.0014	68	7.5	2.57	0.57	0.61
Christmas Island	24	12	0.81±0.07	0.0024±0.0018	18	4.2	2.33	0.55	0.57
Seychelles	48	16	0.61±0.08	0.0019±0.0015	48	6.3	2.51	0.57	0.62

Seventeen sample locations within the Hawaiian Archipelago and nine additional locations across the Indo-Pacific are listed. The number of individuals (*N*) is listed for each marker type. The number of haplotypes (*N*_H_), haplotype diversity (*h*), and nucleotide diversity (π) are listed for cytochrome *b*. Average number of alleles (*A*), allele richness (*A*_R_), observed heterozygosity (*H*_O_), and expected heterozygosity (*H*_E_) are listed for microsatellite loci. Microsatellite data for the Hawaiian Archipelago is based on eleven loci while the Indo-Pacific data is based on eight of these eleven markers (see Table 2). The Hawai'i sample listed under the Indo-Pacific dataset is a random sample of 75 individuals from the larger Hawaiian dataset.

### Mitochondrial cytochrome b

Approximately 560 bp of mitochondrial cytochrome *b* (cyt*b*) were amplified using the primers H15020 [37] and Cyt*b*-07L [38]. Polymerase chain reactions (PCRs) were carried out in a 10 µl volume containing 2–15 ng of template DNA, 0.2–0.3 µM of each primer, 5 µl of the premixed PCR solution BioMix Red™ (Bioline Inc., Springfield, NJ, USA), and deionized water to volume. PCRs utilized the following cycling parameters: initial denaturation at 95°C and final extension at 72°C (10 min each), with an intervening 35 cycles of 30 s at 94°C, 30 s at 48°C, and 45 s at 72°C. Amplification products were purified using 0.75 units of Exonuclease I: 0.5 units of Shrimp Alkaline Phosphatase (ExoSAP; USB, Cleveland, OH, USA) per 7.5 µl PCR products at 37°C for 60 min, followed by deactivation at 80°C for 10 min. DNA sequencing was performed with fluorescently-labeled dideoxy terminators on an ABI 3730XL Genetic Analyzer (Applied Biosystems, Foster City, CA, USA) at the University of Hawai'i Advanced Studies of Genomics, Proteomics and Bioinformatics sequencing facility. Sequences were aligned, edited, and trimmed to a common length using the DNA sequence assembly and analysis software Geneious Pro 5.0 (Biomatters, LTD, Auckland, NZ). In all cases, alignment was unambiguous with no indels or frameshift mutations. Unique haplotypes were identified using the Hapotype Collapser and Converter option in FaBox v.1.35 (http://birc.au.dk/fabox), and deposited in GenBank (accession numbers: JQ083084–JQ083155).

Summary statistics for the mtDNA dataset, including haplotype diversity (*h*) and nucleotide diversity (π), were estimated in arlequin 3.5 [39]. To test for differences in *h* and π between populations we conducted Welch's t-tests, which allow for unequal variances, using the t-test calculator of GraphPad Software (http://www.graphpad.com/quickcalcs/ttest1.cfm). Median-joining networks were constructed using the program network 4.6 with default settings [40]. We calculated the frequency distribution of the number of mutational differences between haplotypes (mismatch analyses), as implemented in arlequin, to determine whether the number of pairwise differences among all DNA sequences reflected expanding or stable populations [41], [42]. To determine confidence intervals around this value we calculated Harpending's raggedness index, r [41] which tests the null hypothesis of an expanding population. This statistic quantifies the smoothness of the observed pairwise mismatch distribution and a non-significant result indicates an expanding population. We also calculated Fu's *F*_S_ [43] using 10,000 permutations which tests for neutrality but is also highly sensitive to population expansions. Significant negative values of *F*_S_ indicate an excess of low-frequency haplotypes, a signature characteristic of either selection or a recent demographic expansion [43].

### Microsatellites

Fifteen microsatellite loci previously designed for *P. filamentosus*, were amplified using the PCR protocols of Gaither et al. [44] with an annealing temperature of 56°C. Two loci (Pfi1.7E and Pfi1.1D) did not reliably amplify specimens and were excluded. Of the 13 loci amplified in the Hawaiian samples, 9 amplified consistently in specimens from across the species range (Table 2). Amplification products were separated on polyacrylamide gels in an ABI 3130XL Genetic Analyzer and scored using genemapper 4.0 with GS500LZ size standards (Applied Biosystems) at the NSF EPSCoR sequencing facility at the Hawai'i Institute of Marine Biology.

**Table 2:** Microsatellite loci used in this study [44].

	All	Hawai'i			Indo-Pacific		
Locus	*N*_A_	*N*_A_	*H*_O_	*H*_E_	*N*_A_	*H*_O_	*H*_E_
*Pfi1.3A*	22	17	0.661	0.682	16	0.472	0.621
Pfi1.5C	14	7	0.520	0.515	14	0.495	0.565
Pfi1.6B2	8	5	0.159	0.157	7	0.252	0.271
Pfi1.9C	13	11	0.668	0.682	11	0.603	0.680
Pfi2.1D	18	13	0.483	0.475	17	0.668	0.706
Pfi2.8A	10	6	0.324	0.332	9	0.286	0.299
Pfi2.8E	13	7	0.517	0.534	11	0.600	0.626
Pfi2.9C	20	12	0.649	0.705	20	0.768	0.848
Pfi4A	32	20	0.543	0.510	29	0.667	0.733
*Pfi1.6B3*	4	4	0.164	0.160			
Pfi2D	3	3	0.189	0.199			
Pfi2.12F	15	15	0.562	0.569			
Pfi2.2E	11	11	0.356	0.357			

Locus name, number of alleles (*N*_A_), observed (*H*_O_), and expected heterozygosity (*H*_E_), are listed for each locus for the Hawai'i samples (*N* = 775, 13 loci) and the Indo-Pacific (*N* = 417, includes the Hawaiian subsample, 9 loci). The number of alleles is also listed for the entire dataset (*N* = 1,117). Loci in italics excluded from analyses due to linkage disequilibrium (see Results).

Most samples were collected by proxy. To avoid using replicate specimens of the same individual we used mstools 3.1 [45] to identify identical genotypes. Microsatellite loci were tested for null alleles, large allele dropout, and scoring errors using micro-checker 2.2 [46]. We tested for departures from Hardy Weinberg equilibrium (HWE) and linkage disequilibrium (LD) using genepop 4.0 [47], [48] and arlequin. We maintained α = 0.05 among all pairwise tests by controlling for the false discovery rate as recommended by Benjamini and Yekutieli [49] and reviewed by Narum [50]. The frequency of null alleles was estimated using FreeNA with 1,000 bootstrap replicates [51]. To test the assumption of neutrality we used the *F*_ST_ outlier method implemented in lositran 1.0 [52], [53]. Observed (*H*_O_) and expected (*H*_E_) heterozygosities, number of alleles, and allelic richness were calculated for each locus per population using fstat 2.9 [54]. Welch's t-tests were used to assess differences in allelic richness between populations as described above.

### Population structure

Population structure was assessed at two geographic scales. First we examined genetic structure among 17 locations in the Hawaiian Archipelago (Fig. 1, Table 1, *N* = 812). Second, we compared samples from nine locations (plus Hawai'i) across the Indo-Pacific (Fig. 1, Table 1, *N* = 485). Due to the large sample size from Hawai'i (*N* = 812) in comparison to other populations across the Indo-Pacific (range *N* = 7–75) we randomly sub-sampled 75 individuals from the Hawaiian dataset for the range-wide comparison. This sub-sample was used in all Indo-Pacific comparisons (Hawai'i, *N* = 75). To test for hierarchical population genetic structure in *P. filamentosus*, an analysis of molecular variance (AMOVA) was performed in arlequin. Two different *F*-statistics were calculated: Wright's *F*_ST_ based on allele frequencies (microsatellites) and an analogue of *F*_ST_ (Φ_ST_), which incorporates the model of sequence evolution (mtDNA). Significance of pairwise *F*-statistics were tested using 20,000 permutations. The AIC implemented in jmodeltest 0.1.1 [55], [56] indicated the TIM+G as the best fit model of cyt*b* sequence evolution. Because this model is not implemented in arlequin, we used the most similar model available [57] with a gamma value of 0.27 to calculate Φ_ST_ values.

Population structure was also assessed using the Bayesian clustering analysis implemented in structure 2.3.3 [58] using sample locations as a prior and the admixture model with correlated allele frequencies. We explored the datasets with initial runs of 1 million steps (burn-in of 20%). We ran 3 replicates for each value of K ranging from 1 to 12 for the Hawaiian dataset and 1 to 10 for the Indo-Pacific dataset. We used structure harvester 0.6.1 to determine which K was the best fit to the data [59] (http://taylor0.biology.ucla.edu/struct_harvest/). Based on the results we conducted an additional 10 replicates for each value of K ranging from 1 to 3 for each dataset.

Mantel tests were performed to determine whether significant isolation-by-distance exists among populations by testing for correlation between pairwise Φ_ST_ (mtDNA) or *F*_ST_ (microsatellites) values and geographic distance using the Isolation-by-Distance Web Service 3.16 [60]. Mantel tests were performed with 20,000 iterations that included negative *F*_ST_/Φ_ST_ values and again with negative *F*_ST_/Φ_ST_ values converted to zeros.

### Migration

Using the results from structure we divided the dataset into K groups, randomly sampled 50 individuals from each group, and estimated long-term average migration rates between groups with migrate 3.1.6 [61], [62]. Estimates of θ (4*N*_e_μ) and M (*m*/μ) were calculated under a Metropolis-Hastings sampling strategy with three replicates of one long MCMC chain run for 1 million generations, a burn-in of 25%, and four short heated chains. Initial runs were conducted with default priors with an unrestricted migration model. Some microsatellite loci consisted of complex repeat motifs and could not be coded by repeat number, therefore, migrate runs for the nuclear dataset were conducted using the infinite allele model of evolution. Posterior distributions for θ and M from initial runs were used to inform priors for subsequent runs. Estimates of the number of migrants between groups per generation (*Nm*) were calculated as θ×M for the mtDNA dataset and as θ×M/4 for the microsatellite dataset, where θ belongs to the recipient population [63].

## Results

Pairs of identical genotypes were detected twice, in specimens from Rowley Shoals and Scotts Reef. Only one of each genotype and the corresponding mtDNA sequence were retained for analyses. Due to geographic proximity and a lack of genetic differentiation (as measured by pairwise Φ_ST_) we grouped the specimens from the Hawaiian locations of Northampton Seamounts and Laysan Island (Northampton), Pioneer and Lisianski (Pioneer), and Maui and Kaho'olawe Islands (Maui).

### Mitochondrial cytochrome b

**Figure 2:**
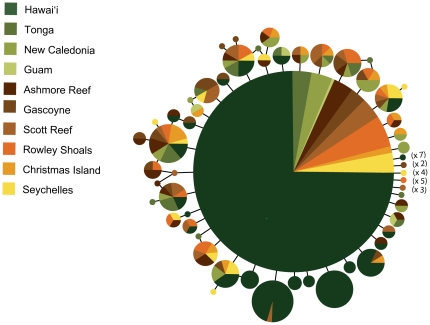
Median-joining network for *Pristipomoides filamentosus*. Network was constructed using the program network 4.6 [40] using 503 bp of cytochrome *b* from 1,202 individuals. Each circle represents one haplotype and the area of the circle is proportional to the number of individuals with that particular haplotype and colors represent collection location (see key). The multipliers in parentheses indicate additional singleton haplotypes observed only at the color coded locations.

We resolved a 503 bp segment of cyt*b* in 1,202 individuals yielding 72 haplotypes including 35 singletons (Table 1, Fig. 2). The number of individuals (*N*), number of haplotypes (*N*_H_), haplotype diversity (*h*), and nucleotide diversity (π) for each location are provided in Table 1. Overall nucleotide diversity in *P. filamentosus* was π = 0.0010 while the corresponding haplotype diversity was *h* = 0.38. Across all samples π = 0.0002–0.0024 and *h* = 0.00–0.81. Hawaiian samples (excluding populations *N*≤5) demonstrated significantly lower values of π (range = 0.0002–0.0013, Welch *t*-test: t = 13.6, df = 23, *P*<0.001) and *h* (range = 0.11–0.46, Welch *t*-test: t = 9.9, df = 23, *P*<0.001) compared to other locations in the Indo-Pacific.

The median-joining network for *P. filamentosus* is a star shaped phylogeny with no more than four bp differences between any two haplotypes. The most common haplotype (78.4% of specimens) was found at every location sampled (Fig. 2). Fu's *F*_S_ for the overall dataset was −3.4×10^38^ (*P*<0.001) indicating an excess of low-frequency haplotypes. When the dataset was divided by geographic region (Hawai'i vs. Indo-Pacific) Fu's *F*_S_ was still significant (−3.4×10^38^, *P*<0.001 and −28.0, *P*<0.001, respectively). The mismatch distribution for all datasets was unimodal (Harpending's raggedness index: overall, *r* = 0.070, *P* = 0.09; Hawai'i, *r* = 0.36, *P* = 0.51; Indo-Pacific, *r* = 0.08, *P* = 0.14). Together these data indicate expanding populations. Under the spatial expansion model we found τ = 0.889, θ_0_ = 0.00, and θ_1_ = ∞. Based on an estimated generation time of 7 years [28], [30] and a molecular clock estimate of 1–2% divergence per 10^6^ years between lineages [64]–[66] we calculated a coalescence time of approximately 88 K–180 K y. Initial female effective population size estimate is *N_e_*_f0_ = 0 and current effective population size estimate is *N_e_*_f1_ = ∞. When only the Hawaiian populations (mtDNA, *N* = 798) were considered τ = 0.262, θ_0_ = 0.00, and θ_1_ = ∞ with a coalescence time of roughly 26 K–52 K y, initial female effective populations size estimate is *N_e_*_f0_ = 0 and current effective population size estimate is *N_e_*_f1_ = ∞. While estimates for the Indo-Pacific population excluding Hawai'i indicated similar effective population sizes, this group had a much older coalescence (τ = 1.02, θ_0_ = 0.00, and θ_1_ = ∞ with a coalescence time of roughly 100 K–200 K y).

### Microsatellites

After correcting for multiple comparisons, there was evidence of physical linkage among the loci pairs Pfi1.3A /Pfi1.5C and Pfi1.6B2 /Pfi1.6B3 in 16 of 17 and 14 of 17 Hawai'i populations, respectively. Pfi1.3A/Pfi1.5C were also in linkage disequilibrium (LD) in 5 of 9 populations outside of Hawai'i. Removal of one of each loci pair from the dataset did not make substantial difference in *F*_ST_ values or the overall patterns of population structure. Therefore, we excluded the loci Pfi1.6B3 and Pfi1.3A from all analyses. Among the remaining eleven markers the following showed evidence of linkage disequilibrium at only one location: Pfi2.9C/Pfi2D (Scott Reef, *P*<0.001), Pfi2.9C /Pfi1.9C (New Caledonia, *P* = 0.003), Pfri2.8A/Pfi1.5C (St. Rogatien, *P*<0.001). The following samples did not meet HWE expectations at single loci: Scott Reef (Pfi1.9C, *P* = 0.005), Christmas Island (Pfi2.9C, *P* = 0.004), New Caledonia (Pfi1.5C, *P* = 0.008), Tonga (Pfi4A, *P*<0.001), Lana'i (Pfi2.1D, *P* = 0.002). In each case an excess of homozygotes was indicated which could reflect the presence of null alleles. However, FreeNA indicated a low frequency of null alleles across loci (range = 0.00–0.06). Calculating *F*_ST_ values using the corrected allele frequencies we found overlapping 95% confidence intervals for all pairs of corrected and uncorrected values. To ensure that patterns of population structure were not driven by any single locus we calculated *F*-statistics by removing one locus at a time. In no case did the patterns change substantially. Using an *F*_ST_ outlier method we found no evidence of selection at any loci.

All microsatellite loci were polymorphic with the number of alleles per locus ranging from 3–32 (Table 1). Hawaiian samples (excluding samples with *N*≤5) demonstrated significantly lower values of allelic richness (*A*_R_ = 1.92–2.02) and *H*_E_ (0.043–0.060) than the other Indo-Pacific locations (*A*_R_ = 2.15–2.59; *H*_E_ = 0.057–0.067; Welch *t*-test: t = 10.3, df = 23, *P*<0.001; t = 9.67, *P*<0.001, respectively). For unknown reasons microsatellite primers failed to amplify fragments in most of the specimens from Gascoyne. Even after a second extraction of DNA from the original tissue we could not get these specimens to amplify. Here we report the data from the Gascoyne specimens (7 out of 44) that amplified for at least 7 of the 9 microsatellites.

### Population structure

We detected low but significant population structure in *P. filamentosus* across the range (Φ_ST_ = 0.029, *P*<0.001; *F*_ST_ = 0.029, *P*<0.001). However, when only the Hawaiian populations were considered overall population structure was not significant (Table 1, 17 populations, *N* = 810) (Φ_ST_ = −0.00078, *P* = 0.536; *F*_ST_ = −0.00001, *P* = 0.502). Only 9 of 272 pairwise comparisons within the archipelago were significant (Table 3; mtDNA = 2 comparisons, microsatellites = 7 comparisons) and these clustered around adjacent St. Rogatien, Brooks Banks, and Gardner in the middle of the island chain. Treating Hawai'i as a single population (*N* = 75), we found low but significant structure across the Indo-Pacific (Table 1, 10 populations, *N* = 485; Φ_ST_ = 0.006, *P* = 0.016; *F*_ST_ = 0.021, *P*<0.001). Pairwise comparisons indicate that most of the genetic structure is due to the distinct population in Hawai'i (Table 4). This population was significantly different from all other populations in both the mtDNA and microsatellite datasets with low to moderate levels of structure (Φ_ST_ = 0.008–0.132, *F*_ST_ = 0.038–0.087). The only other significant pairwise comparisons were between Seychelles and Tonga (mtDNA, *P* = 0.022) and Seychelles and Scott Reef (microsatellites, *P* = 0.019). When Hawai'i was removed from the analysis overall population structure was no longer significant (Φ_ST_ = 0.001, *P* = 0.29; *F*_ST_ = 0.0001, *P* = 0.43).

**Table 3:** Pairwise *F*-statistics for seventeen populations of *Pristipomoides filamentosus* from Hawai'i.

Location	1	2	3	4	5	6	7	8	9	10	11	12	13	14	15	16	17
1.Hawai'i Island	-	−0.002	−0.002	0.002	−0.003	−0.001	−0.004	0.006	0.050	−0.004	0.000	−0.003	0.004	0.010	−0.011	−0.003	−0.006
2. Maui	−0.006	-	−0.001	−0.001	−0.004	−0.002	−0.007	0.007	0.040	−0.003	0.003	−0.001	0.003	0.006	−0.003	0.000	−0.006
3. Lana'i	0.000	−0.003	-	0.001	−0.001	0.002	−0.004	0.008	0.045	0.000	0.003	0.002	0.005	0.003	−0.003	−0.002	0.000
4. Moloka'i	−0.005	−0.009	−0.004	-	0.001	0.000	−0.006	0.007	0.036	−0.001	0.003	0.006^*^	−0.004	−0.003	0.003	0.002	0.000
5.Penguin Banks	0.003	−0.013	−0.007	−0.014	-	0.002	0.002	0.013	0.053	0.000	0.003	−0.002	0.009	0.006	−0.001	−0.002	−0.014
6.O'ahu	0.001	−0.006	−0.007	−0.009	−0.013	-	−0.005	0.007	0.042	−0.004	0.004^*^	−0.001	0.001	0.006	−0.008	0.002	−0.005
7.Kaua'i	0.004	0.001	−0.009	−0.001	−0.004	−0.005	-	0.018	0.032	−0.005	−0.002	0.001	−0.007	−0.007	−0.002	0.000	−0.002
8.Nihoa	−0.100	−0.103	−0.107	−0.099	−0.096	−0.102	−0.111	-	0.071	0.008	0.018	0.010	0.021	0.030	−0.005	0.004	−0.005
9.Necker	0.376	0.185	0.248	0.218	0.174	0.250	0.140	0.189	-	0.038	0.051	0.049	0.040	0.046	0.066^*^	0.050	0.024
10.St. Rogatien	−0.004	−0.004	−0.002	−0.006	−0.009	−0.005	−0.001	−0.101	0.163	-	0.004^*^	−0.002	0.000	0.007	−0.004	−0.002	−0.012
11.Brooks Banks	0.002	−0.004	−0.003	−0.005	−0.010	−0.006	−0.001	−0.103	0.159	0.002	-	0.004	0.005	0.006	−0.010	0.006^*^	0.007
12.Gardner	−0.001	−0.004	−0.005	−0.008	−0.004	−0.006	−0.004	−0.103	0.159	−0.009	0.004	-	0.009^*^	0.015^*^	−0.007	−0.004	−0.010
13.Raita	0.007	−0.002	−0.010	−0.004	−0.005	−0.009	−0.011	−0.109	0.212	−0.001	−0.004	−0.006	-	0.004	0.000	0.004	0.001
14.Maro	−0.030	−0.024	−0.015	−0.023	−0.014	−0.012	−0.012	−0.104	0.340	−0.022	−0.012	−0.023	−0.008	-	0.001	0.011	0.017
15.North Hampton	0.022	−0.007	−0.002	−0.000	−0.001	−0.012	−0.016	−0.089	0.195	−0.009	−0.004	−0.009	−0.007	0.012	-	−0.005	−0.005
16.Pioneer	0.005	0.007	0.003	0.011	0.023^*^	0.008	−0.001	−0.111	0.412	0.006	0.007	0.010^**^	0.005	−0.000	0.023	-	−0.005
17.Salmon Banks	0.120	0.049	0.069	0.062	0.041	0.071	0.028	−0.069	0.035	0.043	0.056	0.038	0.023	0.065	0.019	0.125	-

Pairwise Φ_ST_ values for cytochrome *b* data are below diagonal and *F*_ST_ for eleven microsatellites are above diagonal. Values in bold are significant: *P<0.05, **P<0.01, ***P<0.001.

**Table 4:** Pairwise *F*-statistics for ten populations of *Pristipomoides filamentosus* from across the Indo-Pacific.

Location	1	2	3	4	5	6	7	8	9	10
1.Hawai'i	-	0.038^***^	0.063^***^	0.077^***^	0.084^***^	0.067^***^	0.084^***^	0.061^***^	0.066^***^	0.087^***^
2.Tonga	0.032^***^	-	−0.004	−0.021	−0.031	0.001	−0.020	−0.006	−0.014	−0.020
3.New Caledonia	0.019^***^	0.005	-	−0.004	−0.002	−0.012	−0.003	−0.002	−0.013	0.000
4.Guam	0.132^*^	−0.011	−0.001	-	0.010	0.014	0.005	0.000	0.002	0.016
5.Ashmore Reef	0.037^***^	0.003	0.007	0.007	-	−0.022	−0.002	−0.006	−0.015	0.004
6. Gascoyne	0.022^**^	−0.007	−0.001	−0.005	−0.003	-	−0.008	−0.007	−0.008	−0.008
7.Scott Reef	0.014^**^	−0.001	0.002	−0.007	0.008	−0.007	-	−0.003	−0.020	0.008^*^
8.Rowley Shoals	0.008^*^	−0.004	0.000	−0.013	0.002	0.010	−0.003	-	−0.020	0.005
9.Christmas Island	0.061^***^	0.008	−0.003	0.003	−0.014	−0.005	0.011	0.008	-	0.000
10.Seychelles	0.023^***^	0.014^*^	0.002	−0.001	0.012	0.002	0.006	0.004	0.003	-

**Figure 3:**
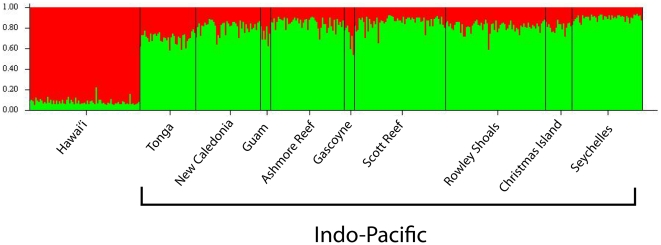
structure plot. The Bayesian clustering analysis of structure 2.3.3 [58] for the Indo-Pacific data resulted in K = 2: Hawai'i vs. all other Indo-Pacific populations (Tonga, New Caledonia, Guam, Ashmore Reef, Gascoyne, Scott Reef, Rowley Shoals, Christmas Island, and Seychelles).

**Figure 4:**
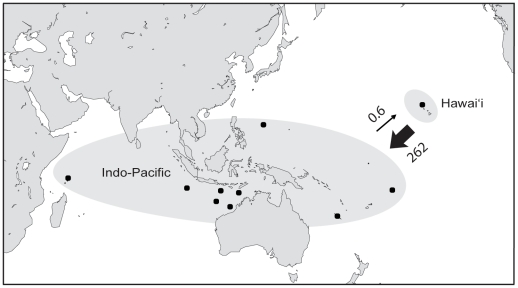
Map depicting migration rates. Migration rates (*Nm*: where *N* is effective female population size and *m* is migration rate) were calculated using migrate 3.1.6 [61], [62]. Locations were grouped according to the results of structure (Fig. 3, K = 2: Hawai'i and the Indo-Pacific. Arrows indicate direction of migration and the size of each arrow is proportional to migration rate. Numbers above the arrows are the number of migrants per generation estimated with mtDNA.

Pairwise Φ_ST_ values for cytochrome *b* data are below diagonal and *F*_ST_ for eight microsatellite loci are above diagonal. Values in bold are significant: *P<0.05, **P<0.01, ***P<0.001.

structure analyses corroborate the patterns of population differentiation revealed with *F*-statistics. No population subdivisions were detected in Hawai'i (K = 1, data not shown), whereas the analysis separated the Indo-Pacific into two populations (K = 2): Hawai'i and the other nine Indo-Pacific sites (Tonga, New Caledonia, Guam, Ashmore Reef, Gascoyne, Scott Reef, Rowley Shoals, Christmas Island, and Seychelles) (Figs. 3, 4). Migration rates estimated from the mitochondrial dataset indicate unidirectional migration between Hawai'i and the Indo-Pacific, with an average of 262 migrants per generation moving from Hawai'i to the west (95% posterior distribution = 46.9–884.4). While only 0.61 migrants per generation was estimated to be making the reverse journey (95% PD = 0–16.9) (Fig. 4). Migration rates estimated from the microsatellite dataset did not corroborate the finding of directional migration detected with the mtDNA [Hawai'i to Indo-Pacific = 42.0 (95% PD = 34.4–50.6); Indo-Pacific to Hawai'i = 49.5 (95% PD = 38.5–60.2)]. There is no evidence for sex biased dispersal in this species and therefore we conclude the discrepancy in migration rates between the mitochondrial and nuclear datasets to be the result of incomplete lineage sorting. It should be noted that migration estimates are long-term averages and as such are probably not precise at ecological time scales.

We found only weak evidence for isolation by distance in the mtDNA: a finding not supported by the microsatellite dataset. Mantel tests showed a correlation between genetic distance (Φ_ST_) and geographic distance across Hawai'i in mtDNA (r = 0.28, *P* = 0.012). However, after excluding Salmon Banks, which is at the furthest end of the sampled range in the archipelago, the correlation was no longer significant (r = 0.06, *P* = 0.244). There was no evidence of isolation by distance across the Indo-Pacific in either the mtDNA or microsatellite datasets.

## Discussion

Our survey of the deepwater snapper *P. filamentosus* revealed significant levels of genetic structure (mtDNA Φ_ST_ = 0.029, microsatellite *F*_ST_ = 0.029) across the Indo-Pacific. However, pairwise population comparisons and the Bayesian clustering analyses indicated that the genetic structure is due to the isolation of Hawai'i. This population is divergent from all other locations in both the mtDNA and nuclear datasets. Overall population structure within Hawai'i was non-significant but we did detect several significant pairwise comparisons that included the adjacent sites of St. Rogatien, Brooks Banks, and Gardner in the middle of the archipelago. Our results indicate that *P. filamentosus* is a highly dispersive species that displays little to no population structuring across 14,000 km from Tonga in the Central Pacific to the Seychelles in the western Indian Ocean, a pattern documented in only two shallow-water reef associated fishes (Bluespine unicornfish, *Naso unicornis* [4]; Bluestripe Snapper, *Lutjanus kasmira* [5]) plus the highly dispersive moray eels [67], [68].

### Population structure in a highly dispersive species

*P. filamentosus* is a deepwater snapper with life history characteristics indicative of high dispersive potential, a hypothesis which is supported by our finding of genetic homogeneity over 14,000 km. This species releases buoyant eggs over a protracted spawning period lasting up to ten months, thus maximizing exposure to the seasonal oceanographic current patterns that transport the early pelagic phases. A pelagic duration lasting up to 180 days allows new recruits to reach considerable size before settlement [26]. Additionally, the documented ability of some mature *P. filamentosus* to disperse 400 km across deep water channels indicates that adults can contribute to dispersal in this species, at least on an archipelagic scale [23].

A lack of population structure across the Central Pacific (exclusive of Hawai'i) has been demonstrated in many shallow-water taxa [2], [5]–[7], [69], [70]. The hundreds of islands and atolls that dot the region between French Polynesia and Australia are thought to act as stepping stones that facilitate dispersal. However, most shallow-water reef taxa show genetic partitions at the IPB around the Sunda and Sahul shelves in the Indo-Malay region (reviewed in [5]). Cessation of dispersal between ocean basins occurred repeatedly during glacial cycles of the Pleistocene and in some cases persisted long enough for populations on either side of the IPB to diverge. The lack of genetic structure across the IPB in *P. filamentosus* could be interpreted as evidence that sea level fluctuations and the corresponding loss of habitat had little effect on this species. Under this scenario some populations of deepwater taxa may have found refugia in the deeper portions of their depth range (below 200 m) so that while the shallow-water fauna underwent widespread extirpations, some deepwater taxa could have persisted and even maintained low levels of gene flow between ocean basins. However, this scenario seems unlikely in *P. filamentosus*. The mtDNA haplotype network for *P. filamentosus* is a tight star shaped phylogeny with haplotypes differing by a maximum of only four mutations and offers no signal of past isolation events. If populations had diverged between oceans during the Pleistocene we would expect a network with greater complexity as detected in a variety of shallow-water taxa [1], [3], [4], [6], [71]. Instead, we observed a pattern more similar to the shallow-water snappers *Lutjanus kasmira* and *L. fulvus* [5]: a tight star shaped mtDNA network that implies a historical bottleneck or a selective sweep that left a single surviving lineage (see [5] for the exception of the Marquesan population). Evidence of an expanding population that coalesces to 100 K–200 K y before present supports this hypothesis.

### Isolation of the Hawaiian Archipelago

The Hawaiian Archipelago is geologically young with Kure, in the northwest, having emerged approximately 30 million years ago [72] (Fig. 1). The geographic isolation of the islands coupled with their young geologic age contributes to the depauperate nature of the Hawaiian fauna and the high level of endemism. Randall [11], [12] records 622 species of shorefishes (<200 m depth) in Hawai'i (compared to 2,700 species in the Indo-Malaysian region) with 25% of these occurring nowhere else. Faunal distributions and oceanographic current patterns indicate that the most likely routes of dispersal into Hawai'i are from southern Japan via the Kuroshio and the North Pacific Current and from the Central Pacific via the Hawaiian Lee Countercurrent (reviewed in [2], [73]). While the fast flowing Northern Equatorial Current would seem to preclude colonization north along the Line Islands 1,400 km south of Hawai'i, the presence of several species (or genetic lineages) in the Line Islands and Hawai'i but not the western Pacific indicates that cross equatorial dispersal occurs [11], [74]. The lack of genetic structure in the Indo-Pacific precludes us from determining the precise route of dispersal into Hawai'i. However, migration rate estimates (mitochondrial dataset) and patterns of genetic structure indicate that contemporary dispersal into the archipelago is extremely low or not occurring. In contrast, average mtDNA migration rate estimates indicate there is a positive flow of migrants out of Hawai'i, as was recently documented in a Hawaiian surgeonfish [73].

Phylogeographic studies reveal isolation of Hawaiian fauna across a diversity of fish and invertebrates including *P. filamentosus* (Table 5). Fourteen of the 18 widely distributed species that have been surveyed showed significant genetic divergence of Hawaiian populations. In the Blueline Surgeonfish, *Acanthurus nigroris*, species level divergence has been detected in Hawai'i (cyt*b* sequence divergence *d* = 4.1%) [75]. Coalescence times reported for Hawaiian populations range from 22 K–45 K y for the Blueline Surgeonfish, *A. nigroris*, to 185 K–371 K y for the Ornate Butterflyfish, *Chaetodon ornatissimus* [8], with *P. filamentosus* near the lower end of the range at 26 K–52 K y (Table 5). Coalescence times do not necessarily indicate founder events in all cases; however the lack of concordance among coalescence times could indicate that colonizations of the Hawaiian Archipelago do not correspond to a single historical or oceanographic event. The fact that three of seven species coalesce to a recent common ancestor between 22 K and 65 K y ago (Table 5) could indicate a glacial-era relaxation of prevailing conditions that inhibit eastward or northward dispersal to Hawai'i.

**Table 5:** Phylogeographic studies of widely distributed marine species.

Species	Marker	Hawai'i distinct?	*F*-statistics/divergence	Coalescence times	Reference
**Fishes**					
*Acanthurus nigroris*	Cyt *b*	Yes	Φ_ST_ = 0.90–0.95	22–45	[75]
*A. nigrofuscus*	Cyt *b*	No	NA	38–117	[7]
*A. triostegus*	allozymes	Yes	*F*_ST_ = 0.24–0.43	NR	[90]
*Zebrasoma flavescens*	Cyt *b*/SSR	Yes	Φ_ST_ = 0.03–0.29*F*_ST_ = 0.08–0.16	130–320	[73]
*Centroypge loriculus*	Cyt *b*	No	NA	NR	[70]
*Chaetodon ornatissimus*	Cyt b/SSR	Yes	Φ_ST_ = 0.11–0.27*F*_ST_ = 0.05–0.16	185–371	[8]
*Chanos chanos*	allozymes	Yes	D = 0.003–0.138	NR	[91]
*Mulloidichthys vanicolensis*	allozymes	Yes	Fixed allele differences	NR	[92]
*Myripristis berndti*	Cyt *b*	No	NA	NR	[2]
*Pristipomoides filamentosus*	Cyt *b*/SSR	Yes	Φ_ST_ = 0.01–0.13*F*_ST_ = 0.04–0.09	26–52	This study
*Chlorurus sordidus*	CR	Yes	2.5%	NR	[1]
*Scarus psittacus*	CR	Yes	Φ_ST_ = 0.06–0.28	65	[93]
*S. rubroviolaceus*	SSR	Yes	*F*_ST_ = 0.105		[69]
*Sphyrna lewini*	Cyt *b*	Yes	Φ_ST_ = 0.17–0.63	280	[94]
*Gymnothorax flavimarginatus*	Cyt *b*/COI/RAG1/RAG2	No	NA	NR	[67], [68]
*G. undulatus*	Cyt *b*/COI/RAG1/RAG2	No	NA	NR	[67], [68]
**Mammals** *Stenella longirostris*	CR/SSR	Yes	Φ_ST_ = 0.22–0.64 *F*_ST_ = 0.06–0.09	NR	[82]
**Sea Cucumbers** *Holothuria atra*	COI	Yes	Φ_ST_ = 0.08–0.89	NR	[74]
**Lobsters** *Panulirus penicillatus*	Cyt *b*	No	NA	NR	Iacchei et al. (unpubl. data)
**Corals** *Montipora capitata*	SSR	Yes	G_ST_ = 0.208–0.490	NR	Concepcion et al. (unpubl. data)

Only studies that included Hawai'i as a sample location are reported. Species, genetic marker, and reference are listed. For species that demonstrate genetically distinct populations at Hawai'i pairwise *F*-statistics (*P*<0.05) or measures of genetic divergences (D = estimate of genetic distance [89]; *d* = sequence divergence) are listed (NA = not applicable). Coalescence times for the Hawaiian populations (×10^3^), if reported, are listed (NR = not reported). Abbreviations for marker types: Cyt *b* = mtDNA cytochrome *b*; CR = mtDNA control region; COI = cytochrome oxidase subunit 1; SSR = simple sequence repeats (microsatellites); RAG = recombination activation gene intron.

### Lower genetic diversity of Hawaiian marine fauna

**Figure 5:**
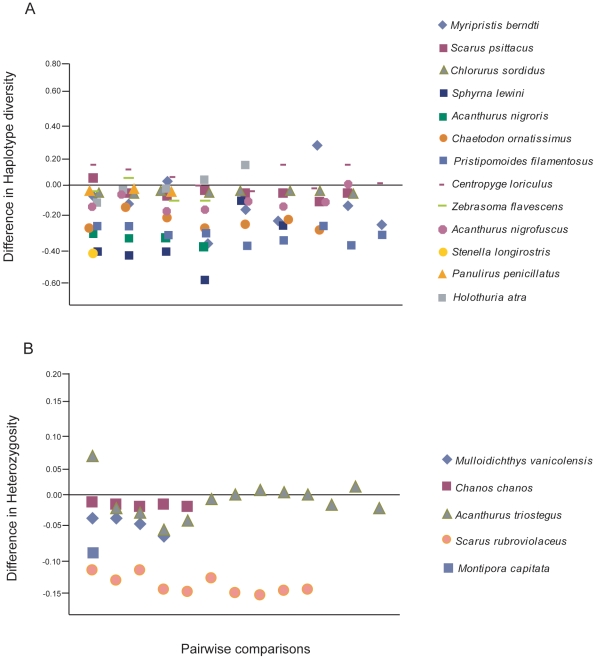
Plots of diversity indices for eighteen widely distributed species of marine organisms. Numbers were calculated by subtracting the A) haplotype diversity (mitochondrial data) or B) expected heterzygosity (microsatellite or allozyme data) of the Hawaiian population from each of up to thirteen populations (X-axis) across the Indo-Pacific. For *Chanos chanos* and *Acanthurus triostegus* indices for multiple Hawaiian populations are reported in the reference and a single index could not be obtained from the authors, therefore, the island of O'ahu was chosen to represent Hawai'i. In the case of *Acanthurus triostegus* the indices reported are observed heterozygosities. Points below the zero line indicate that the Hawaiian population demonstrated lower genetic diversity (96 of 114 comparisons demonstrated lower diversity in Hawai'i; Chi-square = 43.0, df = 1, *P*<0.0001). Only Hawaiian populations of *C. loriculus* and *H. atra* consistently (≥50%) demonstrated higher genetic diversity when compared to populations in the Indo-Pacific. See Table 5 for references.

In nearly 90% of the marine species surveyed (16 of 18), Hawaiian populations demonstrate lower genetic diversity compared to populations in the Central Pacific (Fig. 5). Only the Flame Angelfish, *Centropyge loriculus* [70], and the Common Sea Cucumber, *Holothuria atra* [74], do not show a consistent pattern of lower genetic diversity in Hawai'i. Lower genetic diversity is expected in smaller populations and certainly this may be true for some species in Hawai'i. Alternatively, lower genetic diversity in Hawaiian populations could be an artifact of founder events in which small numbers of individuals colonized the archipelago. Subsequent dispersal events might contribute additional genotypes but if those events are rare then low genetic diversity could be maintained. Examining the data, no consistent pattern arises in support of either hypothesis. However these two scenarios are not mutually exclusive. It is likely that both small effective population sizes in some species as well as the impact of founder events are at work to maintain low genetic diversity in Hawaiian marine populations.

### Population structure within Hawai'i: management implications

*P. filamentosus* constitutes the largest proportion of catch in the commercial bottomfish fishery in Hawai'i [76], [77]. In response to concern over the management of *P. filamentosus*, Shaklee and Samallow [78] conducted a survey of *P. filamentosus* at six locations within Hawai'i using five allozyme markers. They detected no population structure across about two-thirds of the archipelago. Here we increase the geographic coverage to include 17 locations from the Big Island to Salmon Banks at the Northwest end of the Island chain and employ 11 microsatellites. Overall we found no evidence of genetic structure within the Hawaiian Archipelago, as is the case for many shallow-water reef fauna [79]–[81], but for exceptions see [74], [82]. However, we did detect several significant pairwise comparisons that cluster around St. Rogatien, Brooks Banks, and Gardner in the center of the archipelago, a finding similar to patterns observed in the Hawaiian Grouper, *Hyporthodus quernus* (previously *Epinephelus quernus*) [83]–[85]. How can fishery scientists manage a resource like Hawaiian *P. filamentosus* when there appears to be a single genetic stock at the ends of the range (Main Hawaiian Islands and upper NW Hawaiian Islands), but possibly a separate stock in the middle? Seven of the eight significant pairwise comparisons involve St. Rogatien, Brooks Banks, and Gardner Atoll in the middle of the archipelago (RO, BB, and GA in Fig. 1). We are skeptical that strong isolation occurs here given the dispersive nature of this fish. Instead we provisionally support the hypothesis that these significant *F*_ST_ values indicate not an isolated stock, but input from the Johnston Atoll, the only island outside the archipelago that is regarded as part of the Hawaiian biogeographic province based on faunal similarities [86], [87]. Under this assumption, the archipelago should be managed as a single stock. Certainly the part of the Hawaiian range that is currently fished (Kaua'i to Hawai'i Island) is part of a single stock under all lines of evidence. However, even this simple finding carries a confounding caveat. All the genetic surveys to date that have calculated directional migration (see [63]) in Hawaiian marine fauna find that the movement of larvae is from the Main Hawaiian Islands towards the protected NW Hawaiian Islands [73], [88], consistent with prevailing currents. As noted by Toonen et al. [88], the protection of the Papahānaumokuākea Marine National Monument does not alleviate the need to responsibly manage the reefs and resources of the Main Hawaiian Islands.

### Conclusions

Given the sea level changes that accompany glacial cycles and disrupt reef habitats <130 m deep, we expected to find that this deepwater snapper had older, more stable, and more diverse populations than shallow-water fishes. This genetic survey reveals the opposite. We find it remarkable that a snapper with broad depth preference and a vast geographic range coalesces to a common ancestor within the last 100 K–200 K y indicating historical population reductions or a selective sweep during Pleistocene sea level fluctuations. We found evidence of recent population expansion and a lack of population structure across a geographic scale rarely seen in coastal marine fishes. If oceanographic currents in the past are similar to contemporary conditions then it is not just geographic distance isolating Hawai'i. Instead prevailing patterns of larval dispersal indicate that it is much more difficult to get to Hawai'i than to leave. Despite a long early pelagic phase, an ability to disperse as adults, and evidence of extensive gene flow across the Indo-Pacific, *P. filamentosus* is unable to overcome the formidable barriers that isolate Hawai'i from the rest of the Indo-Pacific.
